# Prognostic Value of Platelet-Albumin-Bilirubin Grade in Child-Pugh A and B Patients With Hepatocellular Carcinoma: A Meta-Analysis

**DOI:** 10.3389/fonc.2022.914997

**Published:** 2022-07-13

**Authors:** Rongqiang Liu, Rongqi Li, Min Zhang, Wenbin Liu, Hui Li, Dewei Li

**Affiliations:** ^1^ Department of Hepatobiliary Pancreatic Tumor Center, Chongqing University Cancer Hospital, Chongqing, China; ^2^ Department of Hepatobiliary Surgery, Renmin Hospital of Wuhan University, Wuhan, China; ^3^ Department of Hepatobiliary Surgery, Foshan Hospital of Traditional Chinese Medical, Foshan, China; ^4^ Department of Anesthesiology, Jiulongpo People’s Hospital, Chongqing, China

**Keywords:** hepatocellular carcinoma, platelet-albumin-bilirubin, prognosis, meta-analysis, survival outcome

## Abstract

**Background:**

Numerous studies showed that preoperative platelet-albumin-bilirubin (PALBI) grade was closely related to the prognostic outcome of patients with hepatocellular carcinoma (HCC). However, the conclusions were inconsistent. Therefore, we implemented the study to comprehensively evaluate the association between PALBI grade and prognosis in patients with HCC.

**Methods:**

Relevant articles were collected from the specified databases until February 10, 2022. We included all studies exploring the relationship between PALBI grade and prognosis in HCC patients. We used the hazard ratio (HR) and 95% confidence interval (CI) to calculate the comprehensive analysis. All data analyses were performed using STATA 12.0.

**Results:**

Thirteen retrospective articles containing 15534 patients were included in the meta-analysis. The pooled results displayed that the high PALBI grade was obviously correlated with poor overall survival (OS) (HR: 1.71, 95% CI: 1.46-2.02) and disease-free survival/relapse-free survival (DFS/RFS) (HR:1.31; 95% CI: 1.11–1.54). Subgroup analyses further confirmed the reliability of the comprehensive results.

**Conclusions:**

PALBI may be a valid prognostic indicator in HCC patients. More investigations were needed to test our findings.

## Introduction

Hepatocellular carcinoma (HCC) as one of the most common malignant tumors, seriously endangers human health (1). Studies have revealed that the incidence of HCC will continue to rise (2). Due to many hepatitis B patients in China, it was estimated that China accounted for half of the world’s new HCC patients and deaths each year (3). The 5-year survival rate of early-stage HCC could reach 70% after aggressive treatment (4). However, the survival outcome of patients with advanced HCC was very unsatisfactory, and the five-year survival rate was less than 2% (5). Many prognostic markers have been applied in HCC, but the results are not satisfactory. Therefore, it is particularly necessary to search for novel prognostic markers.

The lifespan of HCC patients is not only related to tumor burden, but also closely contacts with liver function (6). A new assessment model for liver function, called the albumin-bilirubin (ALBI) grade, was introduced by Johnson in 2015 (7). ALBI grade can effectively reflect the liver function and may predict the prognosis of HCC patients (8, 9). Subsequently, a meta-analysis found that the ALBI grade had good diagnostic value in predicting post-hepatectomy liver failure in HCC patients undergoing liver resection (10). However, the ALBI grade did not reflect portal hypertension. To better describe the liver function of HCC patients, a new indicator called PALBI based on the ALBI grade was created (11). PALBI included platelets, and could reflect portal hypertension to some extent. Accumulated studies explored the predictive effect of PALBI grade in HCC patients (12–23). However, their results were inconsistent. Therefore, it was necessary to further clarify the prognostic value of PALBI grade in HCC patients through a comprehensive analysis.

## Materials and Methods

### Search Strategy

The specified databases (PubMed, Embase and Web of Science) were synthetically searched for relevant studies using the terms: “platelet-albumin-bilirubin OR PALBI” AND “hepatocellular carcinoma OR hepatic carcinoma OR hepatoma OR liver cancer OR HCC”. The search deadline was February 10, 2022. Language restrictions did not exist. We also manually checked the included literature references.

### Study Selection

Related literatures were assessed independently by three researchers (Rongqi Li, Rongqiang Liu and Min Zhang). The included studies were identified through discussion. The following criteria were used to select studies: (1) they assessed the prognostic outcome of PALBI grade in HCC patients; (2) there was sufficient data to calculate the hazard ratio (HR) with 95% confidence interval (95% CI). The exclusion criteria were: (1) there was no insufficient data to calculate the HR and 95% CI; (2) they were case abstract, case reports, conference papers, reviews, letters and duplicated studies; and (3) they were animals or cell line studies.

### Data Extraction and Quality Assessment

Some relevant information was extracted, such as author name, publication year, country, study design, sample size, overall survival (OS), disease-free survival/relapse-free survival (DFS/RFS), and HR with 95% CI. Since the results of multivariate analyses were more valuable, we prioritized multivariate analyses. The Newcastle–Ottawa Quality Assessment Scale (NOS) was applied to assess the article quality (24). The study did not need approval from the ethics committee.

### Data Analysis

The pooled data was analyzed by the HR and 95% CI. The forest plot was applied to assess the relationship between PALBI grade and survival outcome in HCC patients. The Engauge Digitizer version 4.1 was used to extract data from graphical survival plots (25). Heterogeneity was analyzed using either the chi-square test or the Cochrane-Q test. P-value <0.05 or I^2^>50% was considered as obvious heterogeneity, and the random-effects model was used. Otherwise, the fixed effects model was applied. Subgroup analyses were carried out to seek heterogeneity sources. Sensitivity analysis was applied to evaluate the stability of the results. Begg’s test and Egger’s test were used to analyze the publication bias. All data analyses were performed using STATA 12.0 (Stata Corporation, College Station, TX, USA). P <0.05 indicated obviously statistical significance.

## Results

### Search Results

**Figure 1:**
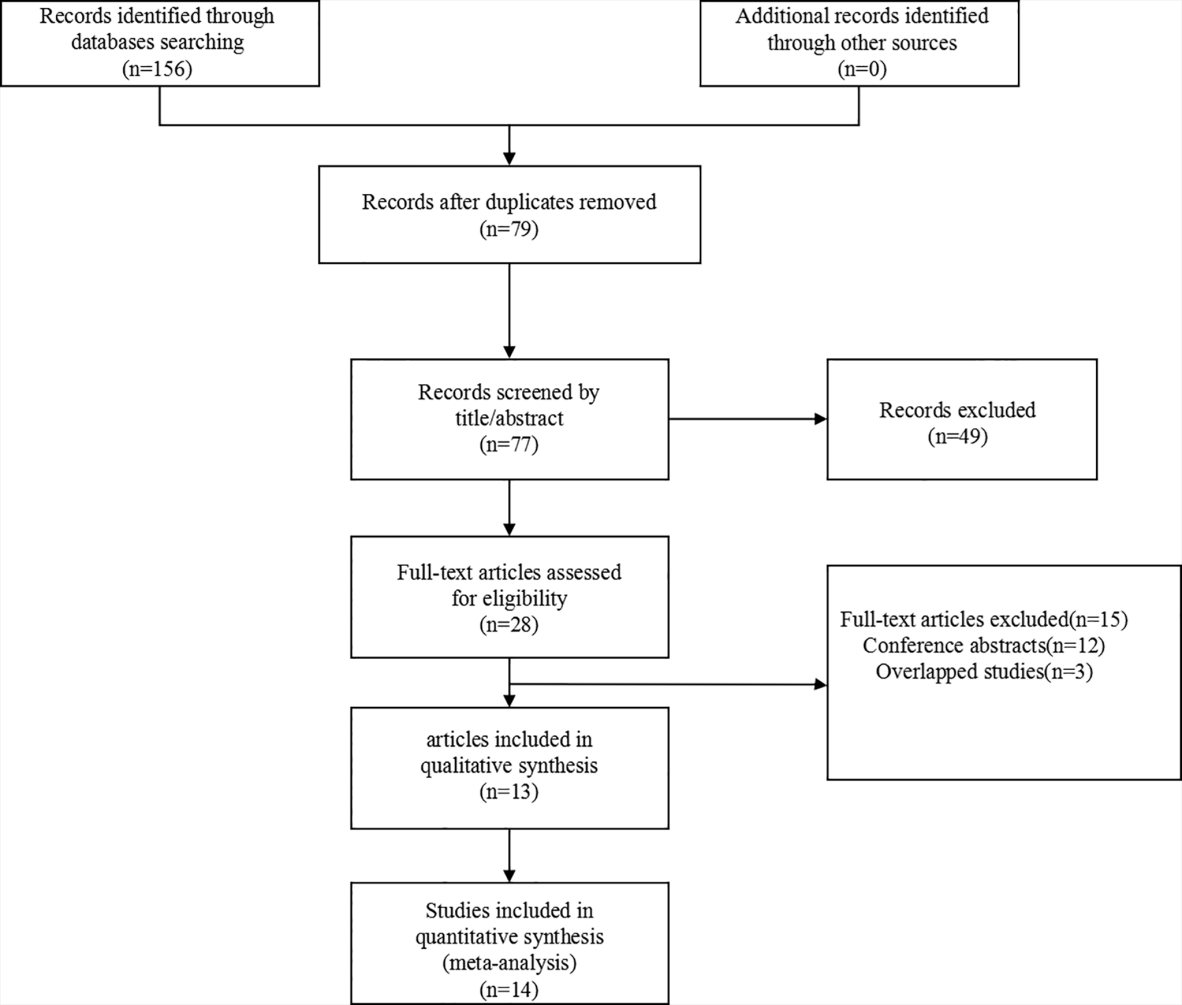
Flow chart of the literature search.

156 articles were initially collected by searching the designated databases. 79 duplicate documents were deleted, leaving 77 articles. After screening the title and abstract, 49 articles were further excluded. After reading the full text, thirteen articles published between 2007 and 2020 were included in the final analysis. The study flow chart was displayed in **Figure 1**.

### Study Characteristics

All articles included in the study were retrospective studies. Four studies were implemented in Europe and America, and ten in Asia. The total number of enrolled patients was 15534. Fourteen studies described OS data, and five reported DFS/RFS data. The quality of each article ranged from 6 to 7 with a mean NOS score of 6.78. Basic information was summarized in **Table 1**.

**Table 1:** Basic information of included studies.

Study	Year	Country	Study type	0ptimal cut-off value of PALBI	Sample size	Treatment methods	Analysis type	Survival analysis	Source of HR	NOS score
Božin	2018	Croatia	retrospective	-2.09	38	With‐surgery	Univariate	OS	Reported	6
Carling	2018	Norway	retrospective	-2.09	49	TACE	Univariate	OS	SC	7
Hansmann	2017	USA	retrospective	-2.09	180	TACE	Univariate	OS	SC	6
Ho	2018A	China	retrospective	-2.09	645	With‐surgery	Univariate	OS、RFS	SC	7
Ho	2018B	China	retrospective	-2.09	174	IHRT	Multivariate	OS	Reported	6
Huang	2020	China	retrospective	-2.09	86	TACE	Univariate	OS	SC	7
Jaruvongvanich	2018	USA	retrospective	-2.09	900	Mixed	Univariate	OS、DFS	SC	7
Lee	2019	Korea	retrospective	-2.09	6669	Mixed	Univariate	OS	SC	7
Liu	2017	China	retrospective	-2.09	3182	Mixed	Univariate	OS	SC	7
Lu	2019	China	retrospective	-2.09	2038	With‐surgery	Univariate	OS	SC	7
Luo	2018	China	retrospective	-2.09	785	With‐surgery	Multivariate	OS、RFS	Reported	7
Ni	2019	China	retrospective	-2.09	349	TACE	Univariate	OS	SC	7
Sonohara	2019	Japan	retrospective	-2.09	305	With‐surgery	Univariate	OS、RFS	Reported	7
Wu	2019	China	retrospective	-2.09	134	With‐surgery	Univariate	OS、RFS	Reported	7

TACE, transcatheter arterial chemoembolization; IHRT, individualized hypofractionated radiotherapy; OS, overall survival; DFS,disease-free survival; RFS, recurrent-free survival; SC, survival curve.

### Association Between High PALBI Grade and OS

**Figure 2:**
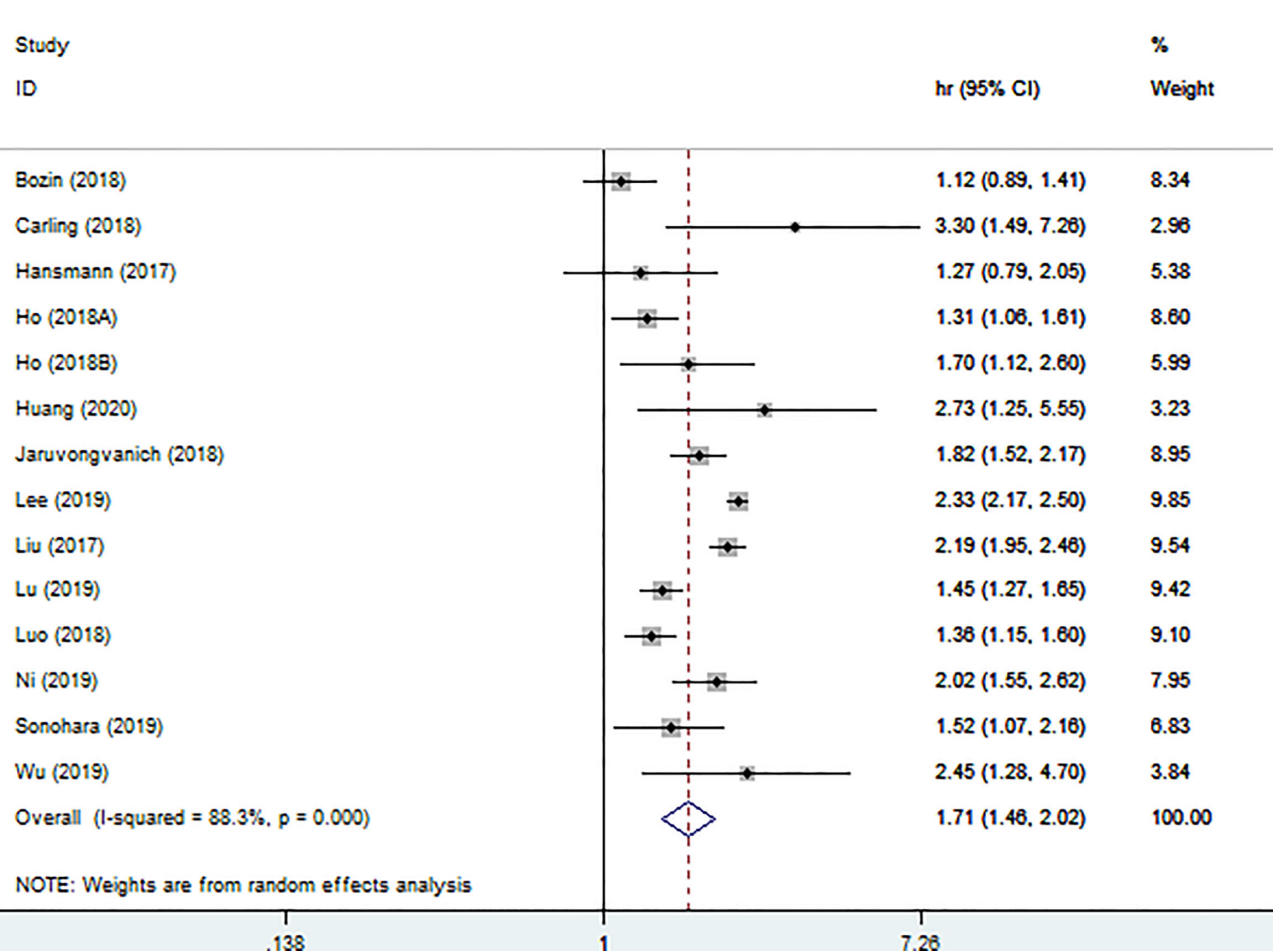
Forest plot of the association between high PALBI grade and OS.

We conducted a meta-analysis using the random effects model because of the significant heterogeneity (I^2 =^ 88.3%). The results demonstrated that high PALBI grade was obviously correlated with worse OS (HR:1.71, 95% CI: 1.46-2.02). The forest plot was described in **Figure 2**.

### Subgroup and Regression Analysis

**Figure 3:**
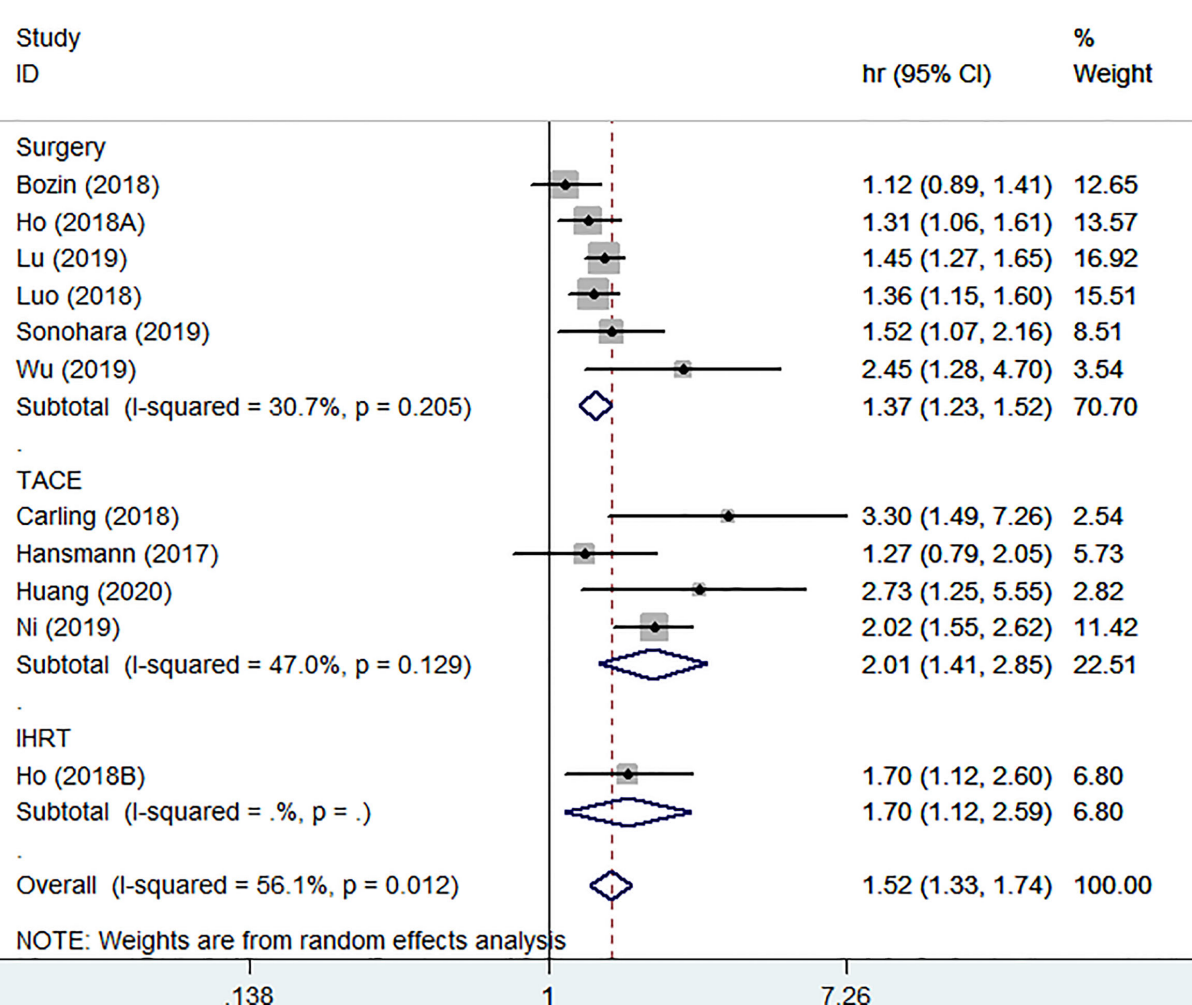
Forest plot of subgroup analysis based on treatment method.

To further explore the heterogeneity sources, we performed subgroup and regression analyses based on treatment method, sample size, analysis type, source of HR and race (**Table 2**). In the subgroup of treatment method, all results showed high PALBI grade presented adverse OS in HCC patients (**Figure 3**). Other subgroup analyses showed surprising consistency. In addition, we also noted that treatment method may be the source of heterogeneity through the meta-regression (P<0.01).

**Table 2:** Subgroups analysis for the prognostic value of high PALBI grade in HCC patients .

Variables	No. of studies	Estimate HR (95%)	*p* value	Heterogeneity		Meta-regression		
				I^2^ (%)	p value	tau^2^	Adj R^2^ (%)	p value
**Treatment**						0.01	81.51	<0.01
Surgery	6	1.37 (1.23-1.52)	<0.01	30.7	0.21			
TACE	4	2.01 (1.41-2.85)	<0.01	47	0.13			
Mixed	3	2.15 (1.91-2.43)	<0.01	69.5	0.04			
IHRT	1	1.70 (1.12-2.59)	0.01	–	–			
**Sample size**						0.06	-11.28	0.99
≥500	6	1.71 (1.38-2.12)	<0.01	93.8	<0.01			
<500	8	1.72 (1.34-2.22)	<0.01	64.9	<0.01			
**Analysis type**						0.56	-3.39	0.44
Univariate analysis	12	1.76 (1.48-2.09)	<0.01	88.1	<0.01			
Multivariate analysis	2	1.40 (1.20-1.63)	<0.01	0	0.33			
**Source of HR**						0.04	22.43	0.13
Reported	5	1.41 (1.17-1.70)	<0.01	45.2	0.12			
SC	9	1.85 (1.54-2.21)	<0.01	87.8	<0.01			
**Race**						0.06	-1.02	0.43
Caucasian	4	1.56 (1.09-2.25)	0.02	79.8	<0.01			
Asian	10	1.78 (1.48-2.13)	<0.01	89.3	<0.01			

TACE, transcatheter arterial chemoembolization; IHRT, Individualized hypofractionated radiotherapy.

### Association Between High PALBI Grade and DFS/RFS

**Figure 4:**
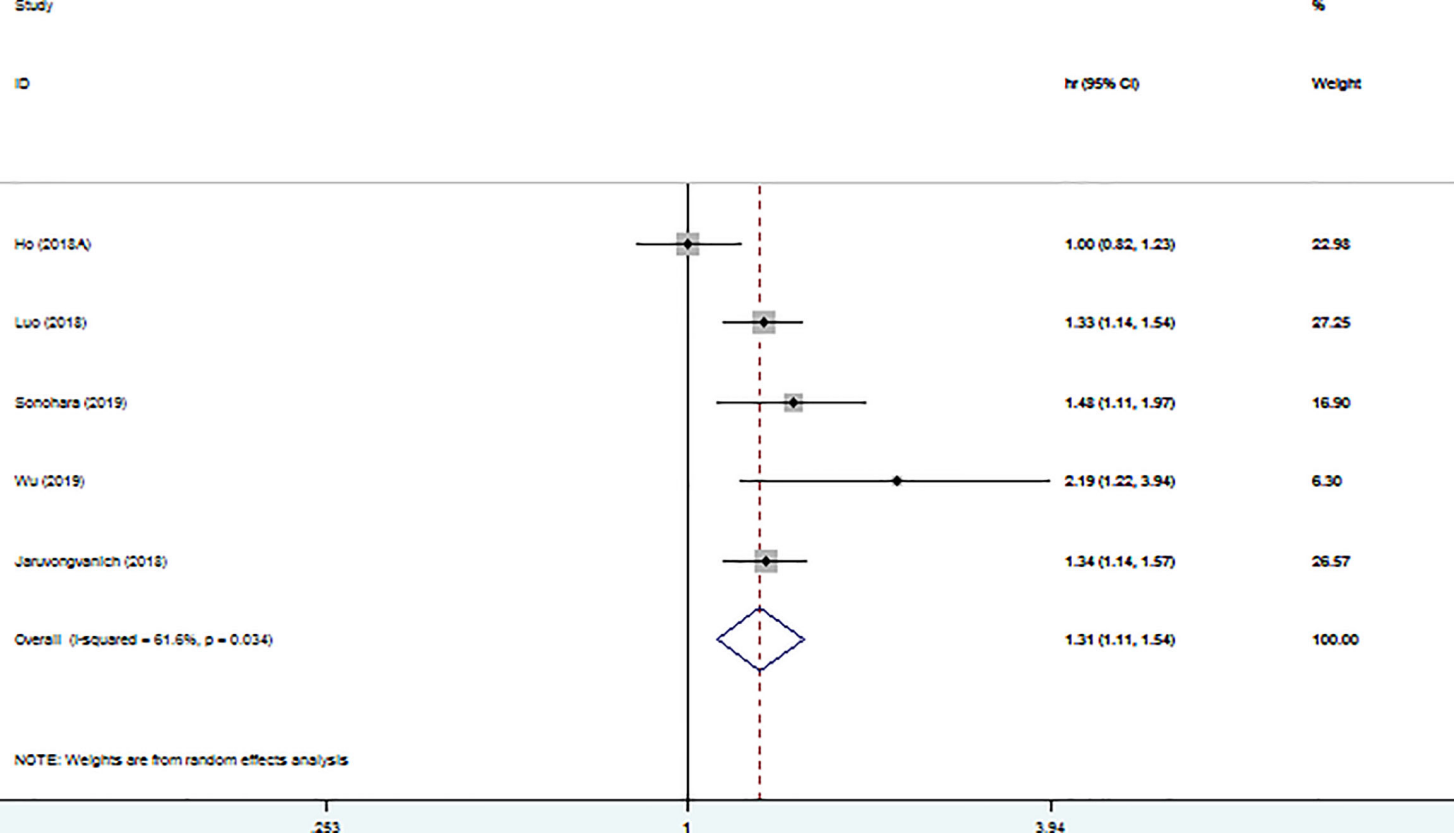
Forest plot of the association between high PALBI grade and DFS/RFS.

Five studies used DFS/RFS to explore the association between PALBI grade and prognosis in HCC patients (**Figure 4**). We found that high PALBI grade was obviously related to unfavorable DFS/RFS (HR: 1.31; 95% CI: 1.11–1.54).

### Sensitivity Analysis

**Figure 5:**
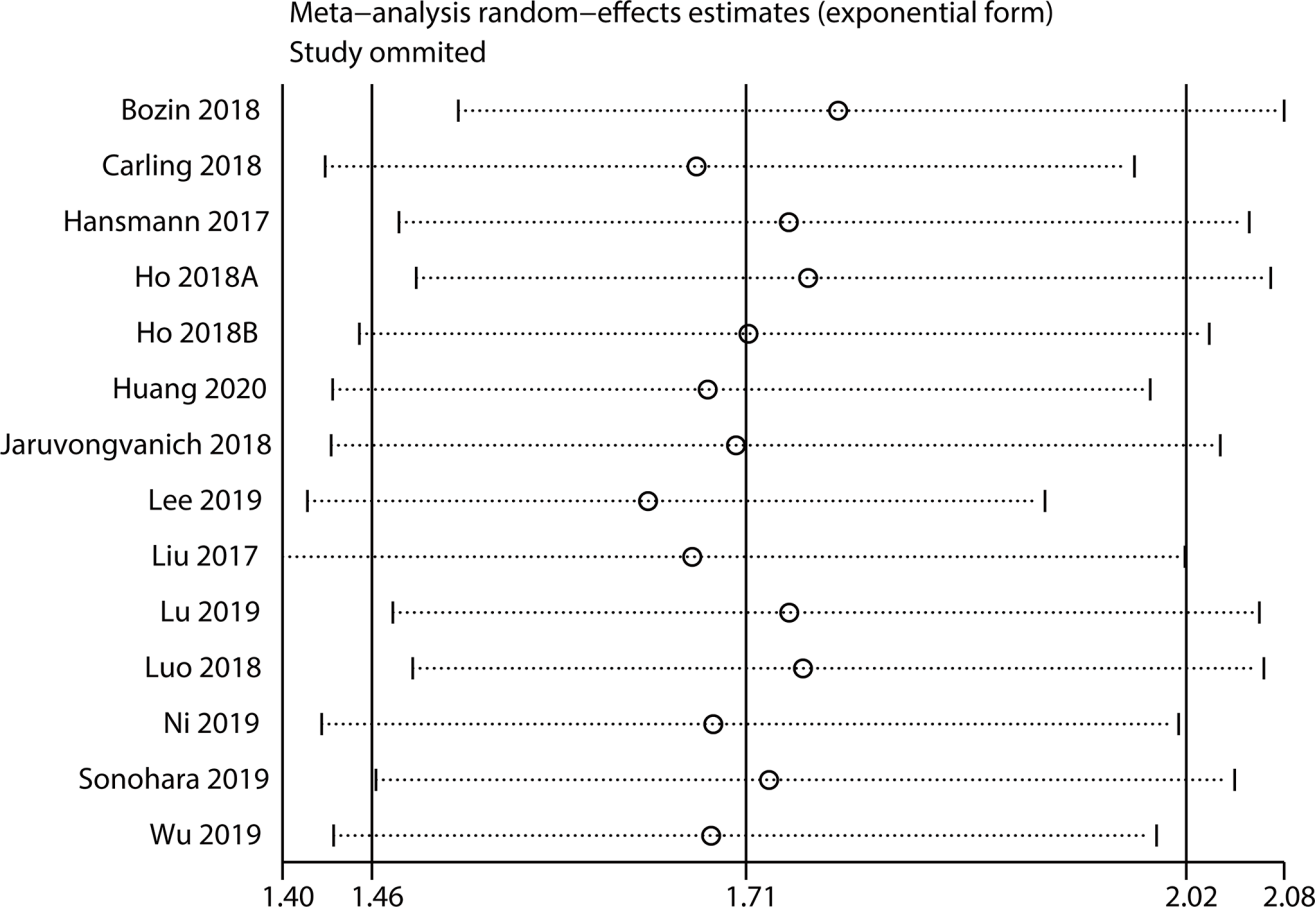
Sensitivity analysis for OS.

**Figure 6:**
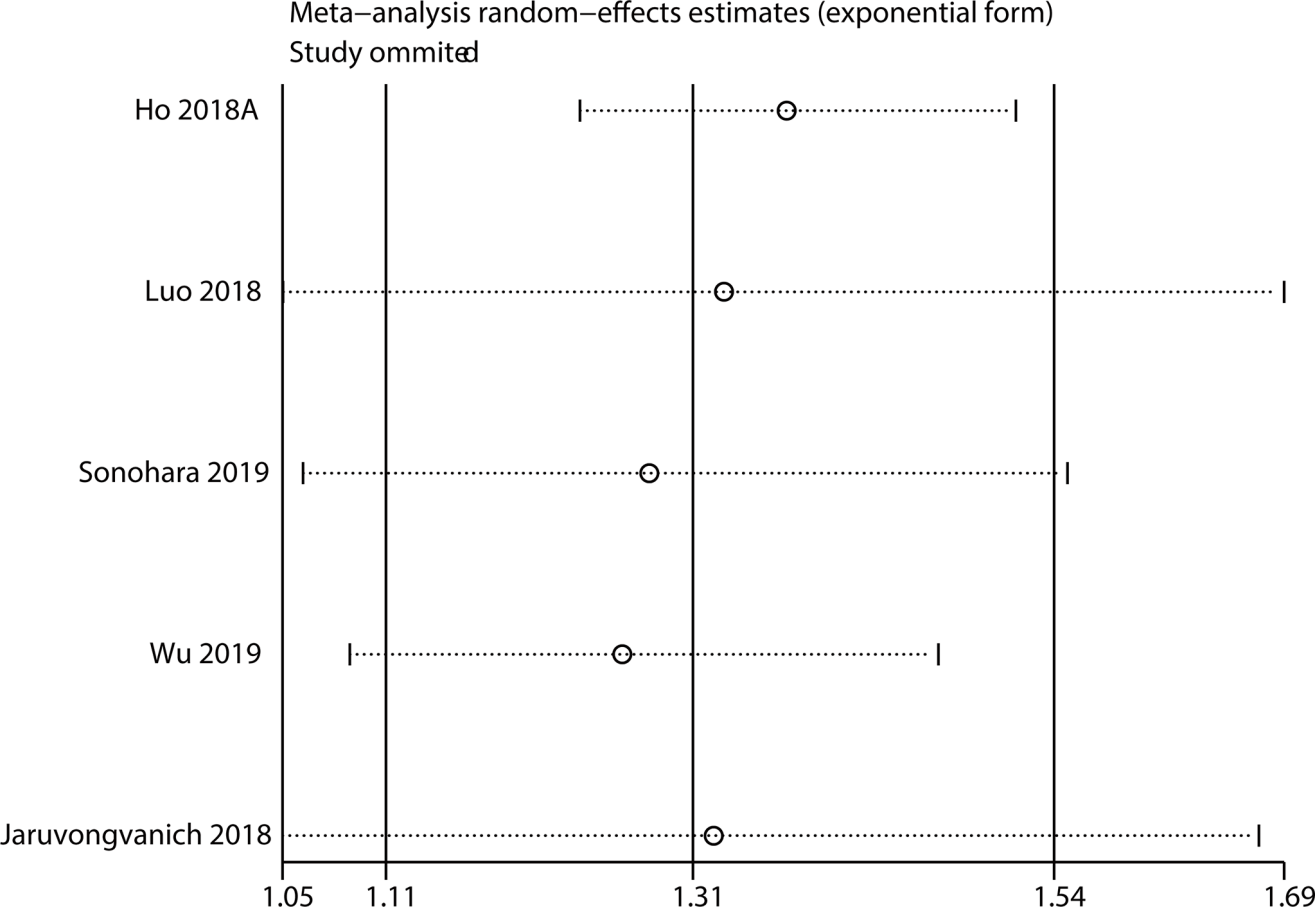
Sensitivity analysis for DFS/PFS.

Sensitivity analysis was implemented to check the stability of the results. The results for OS (**Figure 5**) and DFS/RFS (**Figure 6**) revealed that the outcomes were reliable.

### Publication Bias

**Figure 7:**
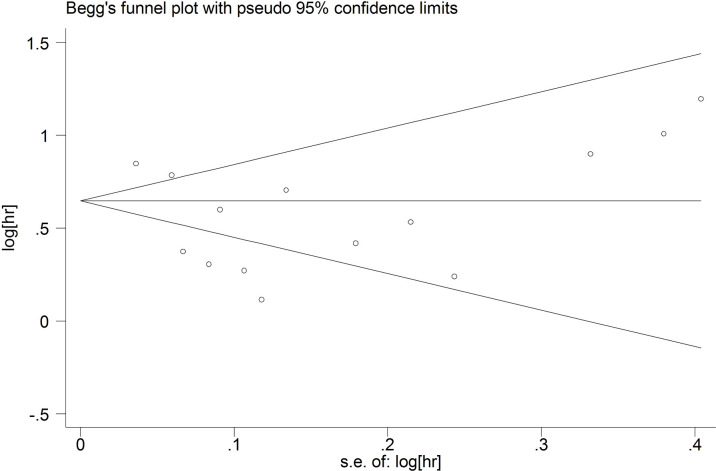
Publication bias for OS.

**Figure 8:**
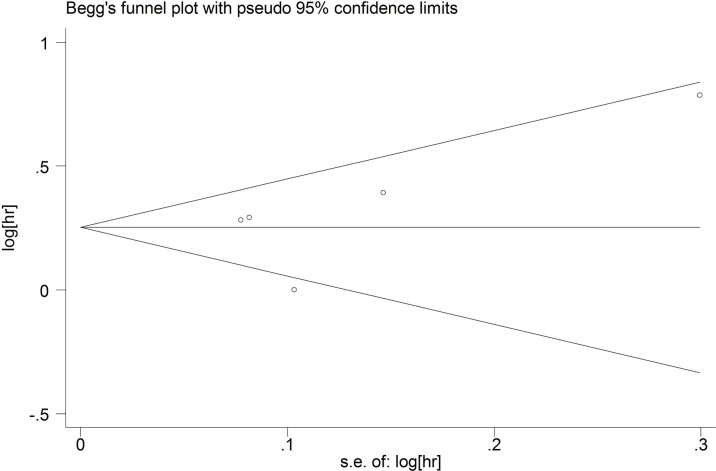
Publication bias for DFS/PFS.

Begg’s test and Egger’s test were used to explore the publication bias. The funnel plots were almost symmetrical. The p values of Begg’s test and Egger’s test for OS were 0.443 and 0.174 (**Figure 7**), respectively. The p values of Begg’s test and Egger’s test for DFS/RFS were 0.221 and 0.450(**Figure 8**), respectively. P values were more than 0.05, and there was no significant bias.

## Discussion

Liver function not only influences the treatment choice for HCC patients but also affects the prognostic outcome of HCC patients. Therefore, the effective evaluation of liver function is crucial for the management for HCC patients. Currently, the Child Turcotte-Pugh (CTP) classification system is the most commonly used indicator to assess liver function. After decades of practice, it has been shown that the Child-Pugh score is not very satisfactory (26). The Model for End-Stage Liver Disease (MELD) score is mainly applied to guide the evaluation of liver transplantation, but rarely used to evaluate the liver function and prognosis of HCC patients (27). In order to further better assess the liver function of HCC patients, PALBI grade was proposed by scholars. As far as we known, the meta-analysis was the first study to systematically examine the prognostic value of PALBI grade in HCC patients. The principal findings revealed that high PALBI grade was obviously associated with worse OS and DFS/RFS. Surprisingly, all subgroup analyses showed that there was obvious correlation between high PALBI grade and unfavorable OS, further confirming the reliability of the comprehensive results.

In our current analysis, patients with different liver function status were divided into high and low risk groups based on PALBI grade. The results showed that regardless of undergoing different treatments, such as surgery, transcatheter arterial chemoembolization(TACE) and mixed, patients in the high PALBI grade group had significantly unfavorable OS compared with those in the low risk group. PALBI grade also showed a good predictive value in advanced HCC patients treated with local chemotherapy in the study. However, no relevant studies had evaluated the predictive effect of PALBI grade in advanced HCC patients with systemic chemotherapy, such as TKIs or ICIs. More researches were needed to assess potential value of PALBI grade for advanced HCC patients with systemic chemotherapy.

In terms of different races, we observed that PALBI grade showed consistent predictive effect for both Asians and Caucasians, suggesting that PALBI grade may be universal for human. In the included data, the etiologies of HCC patients were mainly hepatitis B, hepatitis C and alcoholic cirrhosis. Therefore, whether PALBI grade had the same predictive value on non-B non-C HCC patients such as non-alcoholic fatty liver HCC remained unknown, and further research was needed to explore the issue.

There were some possible reasons to explain our findings. Platelets can release many inflammatory factors which can affect tumor proliferation, invasion and angiogenesis (28, 29). Studies confirmed that high platelets promoted the development, growth, invasion and metastasis of HCC (30, 31). Inhibition of platelet function could effectively prevent HCC metastasis (32). Albumin can reflect the liver function and nutritional status of patients. Hypoalbuminemia patients with cancers often had poor prognosis (33). Therefore, an important measure for the treatment of cancer patients was to increase the patient’s albumin content. Furthermore, animal experiments revealed that albumin could directly regulate the growth of tumor cells by regulating AFP or acting kinases (34). Bilirubin was synthesized and secreted by liver cells, and its level could effectively reflect the liver function. A retrospective clinical study suggested that high bilirubin level was associated with HCC aggressiveness and was an adverse prognosis factor for HCC patients (35).

This study was not without limitations. Firstly, all included articles were retrospective studies. Secondly, most included studies had small sample sizes, which would inevitably leaded to bias. Thirdly, most of the data in the meta-analysis was from studies performed in the Asian region. Fourthly, we unfortunately could not compare the prognostic value of PALBI with ALBI in this meta-analysis due to the lack of raw data. Finally, we could not assess the relationship between PALBI grade and certain pathological parameters, such as gender, tumor stage, metastasis and differentiation.

This meta-analysis had many advantages. Firstly, we confirmed that high PALBI grade strongly predicted unfavorable OS and DFS/RFS. Secondly, sensitivity analysis revealed that the results were not affected. Thirdly, there was no publication bias. Fourthly, we found that the treatment method contributed to the significant heterogeneity. Last but not least, all subgroup analyses suggested that high PALBI grade displayed unfavorable OS.

## Conclusion

We demonstrated that PALBI may be a valid prognostic indicator in HCC patients. As a novel, simple and inexpensive noninvasive predictive index, the PALBI grade was expected to have considerable clinical application value in the prevention and treatment of HCC patients. More investigations were needed to test our findings before the clinical application of the PALBI grade.

## Data Availability Statement

The original contributions presented in the study are included in the article/supplementary material. Further inquiries can be directed to the corresponding authors.

## Author Contributions

HL and DL equally contributed to the study inception and design. RLiu, RLi and MZ equally contributed to the literature search, analysis and writing of the manuscript. WL contributed to the study supervision. All authors approved the final version of the manuscript.

## Conflict of Interest

The authors declare that the research was conducted in the absence of any commercial or financial relationships that could be construed as a potential conflict of interest.

## Publisher’s Note

All claims expressed in this article are solely those of the authors and do not necessarily represent those of their affiliated organizations, or those of the publisher, the editors and the reviewers. Any product that may be evaluated in this article, or claim that may be made by its manufacturer, is not guaranteed or endorsed by the publisher.
